# Moorland vegetation burning debates should avoid contextomy and anachronism: a comment on Davies *et al*. (2016)

**DOI:** 10.1098/rstb.2016.0432

**Published:** 2016-11-19

**Authors:** Lee E. Brown, Joseph Holden, Sheila M. Palmer

**Affiliations:** School of Geography, University of Leeds, Woodhouse Lane, Leeds LS2 9JT, UK

Davies *et al*. [1] called for informed and unbiased debate into the role of fire in UK peatland and moorland management. This general message is something we wholeheartedly agree with, having seen our research presented in various outlets in both a sensationalist and/or a partisan manner (see [1, table 1]). Regrettably though, Davies *et al*. have made mistakes which we believe have amplified the problems, leading to further less-than-refined media headlines [2]. Here, we detail and correct some of the many examples of what we consider to be misrepresentations of our work by [1], such that future debates in this area can indeed proceed in an informed and unbiased manner.

Taking quotations out of context can distort debates [3], skewing both scientific understanding and media representation. To avoid confusion with our citations in this comment, quotes taken from [1] have the original citation numbers removed. A first example of contextomy within [1, p. 7–8] is where they stated:

Brown *et al.* … gave a relatively thorough overview of the limited existing evidence of the changes that burning can induce in hydrological and aquatic systems. In some places, however, their discussion appears to restate popularly held but unsupported assumptions and to rely heavily on unpublished material. For instance, in the section of their paper concerning fire effects on terrestrial vegetation, they state ‘Burning is considered particularly detrimental to peat-forming *Sphagnum* species’. Although they do acknowledge that there is contradictory evidence in the scientific literature ‘from a small number of experimental burning plots’, the only citation to support the initial assertion is a report by the Royal Society for the Protection of Birds (RSPB) that has not been formally published or, to our knowledge, peer reviewed.

Our review paper [4, p. 1412] actually said the following:

Burning is considered particularly detrimental to peat-forming *Sphagnum* species (Grant *et al*. 2012), although some results from a small number of experimental burning plots have contradicted this suggestion (Lee *et al*. 2013). Thus, the processes for changes in *Sphagnum* cover require study in further detail.

The reasons for [1] criticizing selective elements of Brown *et al.* [4] in such a way are, therefore, unclear; we evidently provided a balanced argument that did not simply restate assumptions but evaluated them against other pieces of published work. It also does not ‘rely heavily on unpublished material’; the work of Grant *et al.* [5] is a formally published document with an ISBN which is freely available online or in print, and the study of Lee *et al.* [6] is published in the *Journal of Applied Ecology*.

In a second example, with reference to Brown *et al.* [4], Davies *et al.* [1, p. 9] stated:

Brown *et al*. also point to government guidelines that ‘recommend against burning into living moss layers’ but then comment that ‘this level of control is not always achievable’. Notwithstanding the fact that the fuel moisture content of moss layers during the legal burning period are often high enough to make deep combustion physically impossible in all but the most severe droughts, there is good evidence that moss consumption during prescribed burns is very limited and that exposure of bare peat is rare.

Yet Davies *et al.* [1] have not suggested: that deep combustion is physically impossible in all cases of prescribed burning; that moss consumption is zero and/or that exposure of bare peat is zero, so our statement that this level of control is not always achievable remains valid. Moreover, Davies *et al.* [1, p. 10] stated subsequently that we were right to point out that burn management is sometimes far from perfect. Thus, it is not clear why the authors have extracted selective quotations and presented them in such a critical light when they appear to agree with us.

A third example of misrepresentation by Davies *et al.* [1, p. 10] is

Brown *et al*. rightly point out that much of our knowledge comes from a single long-term experimental study site (the Hard Hill burning/grazing experiment in Cumbria, UK), but then they seek to suggest (again on the basis of an unpublished RSPB report) that the results from that location are not generalizable as the fires are ‘extremely controlled’; despite the fact that the use of controlled fire is precisely the aim of prescribed burning. As far as we are aware, no data have actually been published on prescribed burning practices at Hard Hill or the behaviour of the fires burnt there. Furthermore, the inference that at all other sites fire conditions are not ‘extremely controlled’ would perhaps imply that moorland managers are either not very good at, or do not care about, adequate fire control.

Yet, we did not say that the fires are extremely controlled, nor is it clear why the authors think that we sought to suggest this. Our paper referred to the experimental plot set-up, where, for example, size, shape and treatments are fixed:

“However, these plots may not be typical of managed burns elsewhere given their extremely controlled nature” [4, p. 1413].

Later, the authors confirmed that they agree with us in this regard:

“Brown *et al*. were right to point out that too much of our knowledge comes from a small number of sites and that experimental treatments may not be representative of the variety of management practices on the ground” [1, p. 12].

Thus, the point of their argument is again unclear. We wish to make it clear that we made no insinuation about the ability of moorland managers when using fire as a management tool, directly or implied, contrary to suggestions of Davies *et al.* [1]. Nor have we manipulated or misinterpreted research due to a pre-determined agenda [1, p. 7]. Such accusations have significant potential to undo our relationships with landowners and gamekeepers, and we think they are unbefitting of publication in a scientific journal.

The criticisms of our review publication [4], rebutted above, immediately precede the subsection ‘Representation of science within the media’ in which Davies *et al.* [1] presented a table with 8 (out of 15) examples related to our EMBER project report [7]. Yet, it is impossible that the content of our 2015 review [4] could have influenced the media headlines associated with Brown *et al.* [7] because the article was published in 2014. This anachronism could be interpreted as a means to undermine the primary research of Brown *et al.* [7], given its juxtaposition alongside criticism of Yallop *et al.* [8] and Douglas *et al.* [9] and their associated media coverage. We question why there is no clear discussion of the relationships between our primary research report [7], the associated press release, subsequent media reports and our later review [4]. For the record, a professional media team managed the release of Brown *et al.* [7] in a controlled and orderly manner. The press release is available (22 September 2016) at: http://tinyurl.com/zlud2fx, so Davies *et al.* [1] could easily have undertaken an evaluation to contrast against their critique of Yallop *et al.* [8] and Douglas *et al.* [9].

Prior to the release of Brown *et al.* [7], we provided secure, embargoed access to the text, summary document and press release to numerous journalists, scientists, bloggers and upland agencies/landowners. Once scientific information such as this reaches the public domain and embargoes are lifted, the ways in which users subsequently choose to interpret and disseminate it is always likely to be beyond the control of academics, as Davies *et al*. [1, p. 10] appreciate. More important is that almost all the contents of Brown *et al.* [7] have since been published in peer-reviewed, open-access journals where readers can evaluate those results fully. While Davies *et al*. [1, p. 7] provided various reasons why they think [7] should have been open to more scrutiny from the outset, they then report one of their own perception studies [1, p. 11] without dealing with these same issues of openness to scrutiny and thereby effectively undermine their position. Specifically, the text lacks information about the participants’ constitution (e.g. gender, age), qualifications, preparation and/or existing knowledge, analytical methods, and there are no figures, tables or statistics. Readers are, therefore, unable to evaluate this study in a meaningful way.

In summary, the above examples suggest to us that the readers of Davies *et al*. [1] have the potential to be misled by issues of contextomy and anachrony. In our opinion, Davies *et al*. [1] has, therefore, added further to the often partisan tone of the debate, which is the opposite of what they have called for.
